# 3-(2-Amino­phenyl­sulfanyl)-1,3-diphenyl­propan-1-one

**DOI:** 10.1107/S1600536811022112

**Published:** 2011-06-18

**Authors:** Marzieh Yaeghoobi, Hamid Khaledi, Zanariah Abdullah, Noorsaadah Abd.Rahman

**Affiliations:** a Department of Chemistry, University of Malaya, 50603 Kuala Lumpur, Malaysia

## Abstract

In the title compound, C_21_H_19_NOS, the three aromatic rings are not coplanar, the dihedral angles between them being 84.75 (7), 88.01 (8) and 8.36 (16)°. In the crystal, two types of C—H⋯ π inter­actions, one of which is weak, and N—H⋯π inter­actions link the mol­ecules into layers parallel to the *ab* plane.

## Related literature

For a similar structure, see: Morgant *et al.* (1996 ▶).
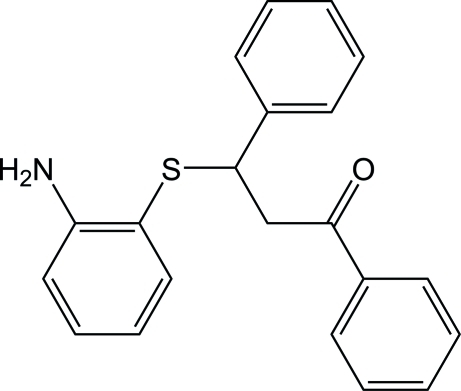


## Experimental

### 

#### Crystal data

C_21_H_19_NOS
*M* *_r_* = 333.43
Monoclinic, 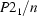

*a* = 11.1741 (16) Å
*b* = 5.6788 (8) Å
*c* = 27.308 (4) Å
β = 95.266 (2)°
*V* = 1725.5 (4) Å^3^
*Z* = 4
Mo *K*α radiation
μ = 0.19 mm^−1^
*T* = 296 K
0.18 × 0.07 × 0.02 mm

#### Data collection

Bruker APEX-II CCD diffractometer
Absorption correction: multi-scan (*SADABS*; Sheldrick, 1996 ▶) *T*_min_ = 0.966, *T*_max_ = 0.996
7303 measured reflections
3195 independent reflections
1784 reflections with *I* > 2σ(*I*)
*R*_int_ = 0.065

#### Refinement

*R*[*F* ^2^ > 2σ(*F* ^2^)] = 0.057
*wR*(*F* ^2^) = 0.101
*S* = 0.95
3195 reflections
223 parameters
H atoms treated by a mixture of independent and constrained refinement
Δρ_max_ = 0.17 e Å^−3^
Δρ_min_ = −0.23 e Å^−3^

### 

Data collection: *APEX2* (Bruker, 2007 ▶); cell refinement: *SAINT* (Bruker, 2007 ▶); data reduction: *SAINT*; program(s) used to solve structure: *SHELXS97* (Sheldrick, 2008 ▶); program(s) used to refine structure: *SHELXL97* (Sheldrick, 2008 ▶); molecular graphics: *X-SEED* (Barbour, 2001 ▶); software used to prepare material for publication: *SHELXL97* and *publCIF* (Westrip, 2010 ▶).

## Supplementary Material

Crystal structure: contains datablock(s) I, New_Global_Publ_Block. DOI: 10.1107/S1600536811022112/go2015sup1.cif

Structure factors: contains datablock(s) I. DOI: 10.1107/S1600536811022112/go2015Isup2.hkl

Additional supplementary materials:  crystallographic information; 3D view; checkCIF report

## Figures and Tables

**Table 1:** Hydrogen-bond geometry (Å, °) *Cg*1 and *Cg*2 are the centroids of the C10–C15 and C16–C21 rings, respectively.

*D*—H⋯*A*	*D*—H	H⋯*A*	*D*⋯*A*	*D*—H⋯*A*
N1—H1*B*⋯*Cg*1^i^	0.91 (3)	2.55 (3)	3.400 (3)	155 (2)
C12—H12⋯*Cg*1^ii^	0.93	2.86	3.581 (4)	135
C14—H14⋯*Cg*2^i^	0.93	2.93	3.587 (3)	128

Symmetry codes: (i) 
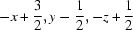
; (ii) 
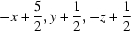
.

**Fig. 1.**
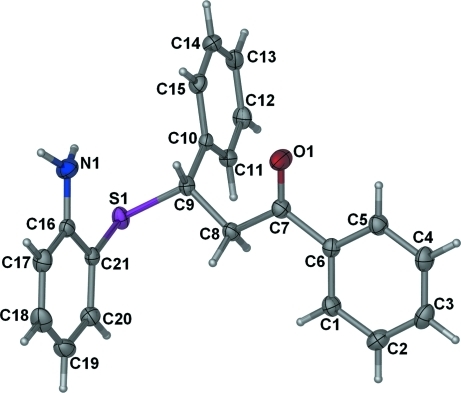
The molecular structure of the title compound with displacement ellipsoids drawn at 30% probability level. Hydrogen atoms are drawn as spheres of arbitrary radius.

**Fig. 2.**
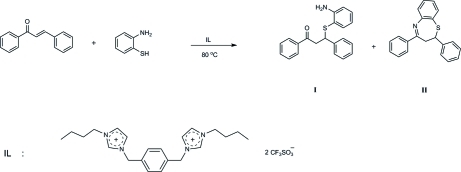
Synthetic route to compounds I and II.

## supplementary crystallographic information

### Comment

The title compound (Fig. 1) was obtained during the synthesis of benzothiazepines II in an ionic liquid media (Fig. 2) and is suggested to be the intermediate compound which upon subsequent cyclization would form the seven-membered thiazepine ring. The three aromatic rings of the molecule are non-planar as are in the structure of a related compound (Morgant *et al.*, 1996). The dihedral angles between the phenyl rings plane are 84.75 (7)° (between C1/C6 and C10/C15), 88.01 (8) ° (between C1/C6 and C16/C21) and 8.36 (16) ° (between C10/C15 and C16/C21). The amino hydrogen, H1B, is involved in an N—H··· π interaction (Table 1). In the crystal, the N—H···π interactions connect the adjacent molecules into infinite chains along the *b* axis. The one-dimensional link is supplemented by weak C14—H14··· π interactions. The chains are connected into two-dimensional-arrays parallel to the *ab* plane *via* C12—H12··· π interactions (Table 1).

### Experimental

The title compound was synthesized as illustrated in Fig. 2. A mixture of the chalcone (0.208 g, 1 mmol), *o*-aminothiophenol (0.088 ml, 1.1 mmol) and ionic liquid, IL, (0.1 g) was heated at 80°C for 80 min. The two products, I & II, were extracted with diethyether and separated by column chromatography (hexane: ethylacatate, 8:2). The second fraction, containing (I), was evaporated and the resulting solid was recrystallized from ethanol at room temperature to give the colorless crystals of the title compound.

### Refinement

The C-bound hydrogen atoms were placed at calculated positions and refined as riding atoms with C—H distances of 0.93 (*phenyl*), 0.97 (*methylene*) and 0.98 (*methine*) Å. The N-bound hydrogen atoms were located in a difference Fourier map and refined freely. For all hydrogen atoms *U*iso(H) were set to1.2 *U*eq(carrier atom).

### Crystal data

**Table:**

C_21_H_19_NOS	*F*(000) = 704
*M**_r_* = 333.43	*D*_x_ = 1.284 Mg m^−^^3^
Monoclinic, *P*2_1_/*n*	Mo *K*α radiation, λ = 0.71073 Å
Hall symbol: -P 2yn	Cell parameters from 713 reflections
*a* = 11.1741 (16) Å	θ = 3.0–20.6°
*b* = 5.6788 (8) Å	µ = 0.19 mm^−^^1^
*c* = 27.308 (4) Å	*T* = 296 K
β = 95.266 (2)°	Lath, colorless
*V* = 1725.5 (4) Å^3^	0.18 × 0.07 × 0.02 mm
*Z* = 4	

### Data collection

**Table:**

Bruker APEX-II CCD diffractometer	3195 independent reflections
Radiation source: fine-focus sealed tube	1784 reflections with *I* > 2σ(*I*)
graphite	*R*_int_ = 0.065
φ and ω scans	θ_max_ = 25.5°, θ_min_ = 2.0°
Absorption correction: multi-scan (*SADABS*; Sheldrick, 1996)	*h* = −12→13
*T*_min_ = 0.966, *T*_max_ = 0.996	*k* = −6→5
7303 measured reflections	*l* = −29→33

### Refinement

**Table:**

Refinement on *F*^2^	Primary atom site location: structure-invariant direct methods
Least-squares matrix: full	Secondary atom site location: difference Fourier map
*R*[*F*^2^ > 2σ(*F*^2^)] = 0.057	Hydrogen site location: inferred from neighbouring sites
*wR*(*F*^2^) = 0.101	H atoms treated by a mixture of independent and constrained refinement
*S* = 0.95	*w* = 1/[σ^2^(*F*_o_^2^) + (0.0336*P*)^2^] where *P* = (*F*_o_^2^ + 2*F*_c_^2^)/3
3195 reflections	(Δ/σ)_max_ < 0.001
223 parameters	Δρ_max_ = 0.17 e Å^−^^3^
0 restraints	Δρ_min_ = −0.23 e Å^−^^3^

### Special details

**Table:**

Geometry. All e.s.d.'s (except the e.s.d. in the dihedral angle between two l.s. planes) are estimated using the full covariance matrix. The cell e.s.d.'s are taken into account individually in the estimation of e.s.d.'s in distances, angles and torsion angles; correlations between e.s.d.'s in cell parameters are only used when they are defined by crystal symmetry. An approximate (isotropic) treatment of cell e.s.d.'s is used for estimating e.s.d.'s involving l.s. planes.
Refinement. Refinement of *F*^2^ against ALL reflections. The weighted *R*-factor *wR* and goodness of fit *S* are based on *F*^2^, conventional *R*-factors *R* are based on *F*, with *F* set to zero for negative *F*^2^. The threshold expression of *F*^2^ > σ(*F*^2^) is used only for calculating *R*-factors(gt) *etc*. and is not relevant to the choice of reflections for refinement. *R*-factors based on *F*^2^ are statistically about twice as large as those based on *F*, and *R*- factors based on ALL data will be even larger.

### Fractional atomic coordinates and isotropic or equivalent isotropic displacement parameters (Å^2^)

**Table:**

	*x*	*y*	*z*	*U*_iso_*/*U*_eq_	
S1	0.80339 (6)	0.87951 (14)	0.18814 (3)	0.0447 (2)	
O1	1.0501 (2)	0.3126 (4)	0.13496 (8)	0.0647 (7)	
N1	0.6375 (2)	0.4530 (5)	0.18086 (10)	0.0544 (8)	
H1A	0.684 (3)	0.492 (5)	0.2078 (11)	0.065*	
H1B	0.576 (3)	0.353 (5)	0.1859 (10)	0.065*	
C1	1.0977 (3)	0.7137 (6)	0.03620 (11)	0.0486 (8)	
H1	1.0485	0.8384	0.0436	0.058*	
C2	1.1576 (3)	0.7203 (6)	−0.00593 (11)	0.0583 (9)	
H2	1.1483	0.8497	−0.0268	0.070*	
C3	1.2307 (3)	0.5382 (7)	−0.01713 (11)	0.0576 (10)	
H3	1.2698	0.5429	−0.0457	0.069*	
C4	1.2458 (3)	0.3503 (6)	0.01372 (11)	0.0567 (9)	
H4	1.2970	0.2285	0.0065	0.068*	
C5	1.1859 (2)	0.3390 (5)	0.05554 (10)	0.0479 (8)	
H5	1.1957	0.2084	0.0761	0.057*	
C6	1.1110 (2)	0.5215 (5)	0.06716 (10)	0.0372 (7)	
C7	1.0446 (3)	0.4961 (6)	0.11240 (11)	0.0421 (8)	
C8	0.9699 (2)	0.7008 (5)	0.12804 (9)	0.0407 (8)	
H8A	0.9049	0.7301	0.1027	0.049*	
H8B	1.0198	0.8409	0.1310	0.049*	
C9	0.9174 (2)	0.6565 (5)	0.17669 (9)	0.0389 (7)	
H9	0.8789	0.5013	0.1753	0.047*	
C10	1.0071 (2)	0.6649 (5)	0.22161 (9)	0.0330 (7)	
C11	1.0841 (2)	0.8534 (5)	0.23096 (10)	0.0397 (7)	
H11	1.0841	0.9755	0.2083	0.048*	
C12	1.1609 (2)	0.8630 (6)	0.27341 (11)	0.0484 (8)	
H12	1.2120	0.9912	0.2792	0.058*	
C13	1.1620 (3)	0.6835 (6)	0.30718 (11)	0.0511 (9)	
H13	1.2142	0.6889	0.3357	0.061*	
C14	1.0852 (3)	0.4954 (6)	0.29848 (11)	0.0502 (9)	
H14	1.0850	0.3743	0.3214	0.060*	
C15	1.0089 (2)	0.4857 (5)	0.25623 (10)	0.0436 (8)	
H15	0.9577	0.3574	0.2507	0.052*	
C16	0.6144 (2)	0.6174 (5)	0.14430 (10)	0.0378 (7)	
C17	0.5196 (3)	0.5843 (6)	0.10811 (11)	0.0507 (9)	
H17	0.4728	0.4490	0.1084	0.061*	
C18	0.4949 (3)	0.7500 (7)	0.07200 (11)	0.0577 (9)	
H18	0.4307	0.7263	0.0483	0.069*	
C19	0.5635 (3)	0.9510 (6)	0.07023 (11)	0.0559 (9)	
H19	0.5454	1.0636	0.0460	0.067*	
C20	0.6588 (3)	0.9817 (6)	0.10486 (10)	0.0465 (8)	
H20	0.7062	1.1157	0.1035	0.056*	
C21	0.6864 (2)	0.8180 (5)	0.14189 (9)	0.0338 (7)	

### Atomic displacement parameters (Å^2^)

**Table:**

	*U*^11^	*U*^22^	*U*^33^	*U*^12^	*U*^13^	*U*^23^
S1	0.0366 (4)	0.0552 (6)	0.0419 (5)	0.0055 (4)	0.0013 (3)	−0.0142 (4)
O1	0.0848 (17)	0.0482 (16)	0.0659 (16)	0.0140 (14)	0.0324 (13)	0.0095 (12)
N1	0.0539 (18)	0.055 (2)	0.0551 (19)	−0.0092 (15)	0.0070 (14)	0.0093 (15)
C1	0.0494 (19)	0.046 (2)	0.051 (2)	0.0072 (17)	0.0098 (16)	−0.0029 (16)
C2	0.066 (2)	0.062 (3)	0.049 (2)	−0.007 (2)	0.0129 (18)	0.0073 (17)
C3	0.056 (2)	0.076 (3)	0.043 (2)	−0.011 (2)	0.0160 (17)	−0.011 (2)
C4	0.054 (2)	0.064 (3)	0.054 (2)	0.005 (2)	0.0158 (17)	−0.0135 (19)
C5	0.0485 (18)	0.048 (2)	0.0475 (19)	0.0047 (17)	0.0076 (15)	−0.0042 (15)
C6	0.0360 (16)	0.041 (2)	0.0340 (17)	−0.0026 (16)	0.0022 (13)	−0.0057 (15)
C7	0.0443 (18)	0.041 (2)	0.0407 (19)	0.0012 (17)	0.0034 (14)	−0.0034 (16)
C8	0.0407 (17)	0.045 (2)	0.0359 (17)	0.0067 (16)	0.0029 (14)	−0.0011 (14)
C9	0.0352 (16)	0.0400 (19)	0.0422 (18)	0.0033 (15)	0.0077 (14)	−0.0058 (14)
C10	0.0278 (15)	0.039 (2)	0.0333 (17)	−0.0005 (15)	0.0076 (12)	−0.0016 (14)
C11	0.0399 (17)	0.040 (2)	0.0396 (18)	0.0015 (17)	0.0046 (14)	0.0029 (14)
C12	0.0359 (17)	0.055 (2)	0.054 (2)	−0.0087 (17)	0.0011 (15)	−0.0067 (18)
C13	0.0375 (18)	0.080 (3)	0.0357 (18)	0.0085 (19)	0.0028 (15)	−0.0006 (19)
C14	0.0458 (19)	0.060 (2)	0.046 (2)	0.0026 (18)	0.0103 (16)	0.0154 (16)
C15	0.0386 (17)	0.043 (2)	0.050 (2)	−0.0052 (16)	0.0071 (16)	0.0022 (16)
C16	0.0350 (16)	0.045 (2)	0.0346 (17)	0.0033 (17)	0.0080 (14)	−0.0009 (15)
C17	0.0433 (18)	0.059 (2)	0.050 (2)	−0.0076 (18)	0.0053 (16)	−0.0097 (18)
C18	0.047 (2)	0.081 (3)	0.043 (2)	0.003 (2)	−0.0101 (16)	−0.0156 (19)
C19	0.062 (2)	0.066 (3)	0.039 (2)	0.015 (2)	0.0013 (17)	0.0046 (16)
C20	0.0484 (19)	0.049 (2)	0.0423 (19)	0.0011 (17)	0.0056 (16)	0.0006 (16)
C21	0.0306 (15)	0.0382 (19)	0.0334 (17)	0.0019 (15)	0.0069 (13)	−0.0054 (14)

### Geometric parameters (Å, °)

**Table:**

S1—C21	1.767 (3)	C9—H9	0.9800
S1—C9	1.843 (3)	C10—C11	1.382 (3)
O1—C7	1.209 (3)	C10—C15	1.388 (4)
N1—C16	1.374 (4)	C11—C12	1.379 (4)
N1—H1A	0.89 (3)	C11—H11	0.9300
N1—H1B	0.91 (3)	C12—C13	1.374 (4)
C1—C6	1.380 (4)	C12—H12	0.9300
C1—C2	1.384 (4)	C13—C14	1.377 (4)
C1—H1	0.9300	C13—H13	0.9300
C2—C3	1.370 (4)	C14—C15	1.371 (4)
C2—H2	0.9300	C14—H14	0.9300
C3—C4	1.360 (4)	C15—H15	0.9300
C3—H3	0.9300	C16—C17	1.393 (4)
C4—C5	1.377 (4)	C16—C21	1.400 (4)
C4—H4	0.9300	C17—C18	1.373 (4)
C5—C6	1.387 (4)	C17—H17	0.9300
C5—H5	0.9300	C18—C19	1.378 (4)
C6—C7	1.505 (4)	C18—H18	0.9300
C7—C8	1.515 (4)	C19—C20	1.369 (4)
C8—C9	1.522 (3)	C19—H19	0.9300
C8—H8A	0.9700	C20—C21	1.387 (4)
C8—H8B	0.9700	C20—H20	0.9300
C9—C10	1.512 (3)		
			
C21—S1—C9	102.73 (12)	C11—C10—C15	118.2 (3)
C16—N1—H1A	119 (2)	C11—C10—C9	121.9 (2)
C16—N1—H1B	116.2 (19)	C15—C10—C9	119.8 (3)
H1A—N1—H1B	115 (3)	C12—C11—C10	121.0 (3)
C6—C1—C2	119.8 (3)	C12—C11—H11	119.5
C6—C1—H1	120.1	C10—C11—H11	119.5
C2—C1—H1	120.1	C13—C12—C11	120.1 (3)
C3—C2—C1	120.6 (3)	C13—C12—H12	120.0
C3—C2—H2	119.7	C11—C12—H12	120.0
C1—C2—H2	119.7	C12—C13—C14	119.5 (3)
C4—C3—C2	119.8 (3)	C12—C13—H13	120.2
C4—C3—H3	120.1	C14—C13—H13	120.2
C2—C3—H3	120.1	C15—C14—C13	120.4 (3)
C3—C4—C5	120.5 (3)	C15—C14—H14	119.8
C3—C4—H4	119.7	C13—C14—H14	119.8
C5—C4—H4	119.7	C14—C15—C10	120.8 (3)
C4—C5—C6	120.3 (3)	C14—C15—H15	119.6
C4—C5—H5	119.9	C10—C15—H15	119.6
C6—C5—H5	119.9	N1—C16—C17	120.2 (3)
C1—C6—C5	118.9 (3)	N1—C16—C21	121.1 (3)
C1—C6—C7	123.0 (3)	C17—C16—C21	118.7 (3)
C5—C6—C7	118.0 (3)	C18—C17—C16	120.5 (3)
O1—C7—C6	119.7 (3)	C18—C17—H17	119.8
O1—C7—C8	121.4 (3)	C16—C17—H17	119.8
C6—C7—C8	118.9 (3)	C17—C18—C19	121.1 (3)
C7—C8—C9	112.7 (2)	C17—C18—H18	119.4
C7—C8—H8A	109.1	C19—C18—H18	119.4
C9—C8—H8A	109.1	C20—C19—C18	118.8 (3)
C7—C8—H8B	109.1	C20—C19—H19	120.6
C9—C8—H8B	109.1	C18—C19—H19	120.6
H8A—C8—H8B	107.8	C19—C20—C21	121.7 (3)
C10—C9—C8	115.0 (2)	C19—C20—H20	119.1
C10—C9—S1	104.94 (17)	C21—C20—H20	119.1
C8—C9—S1	111.25 (19)	C20—C21—C16	119.3 (3)
C10—C9—H9	108.5	C20—C21—S1	119.3 (2)
C8—C9—H9	108.5	C16—C21—S1	121.2 (2)
S1—C9—H9	108.5		

### Hydrogen-bond geometry (Å, °)

**Table:**

Cg1 and Cg2 are the centroids of the C10–C15 and C16–C21 rings, respectively.

**Table:**

*D*—H···*A*	*D*—H	H···*A*	*D*···*A*	*D*—H···*A*
N1—H1B···Cg1^i^	0.91 (3)	2.55 (3)	3.400 (3)	155 (2)
C12—H12···Cg1^ii^	0.93	2.86	3.581 (4)	135
C14—H14···Cg2^i^	0.93	2.93	3.587 (3)	128

Symmetry codes: (i) −*x*+3/2, *y*−1/2, −*z*+1/2; (ii) −*x*+5/2, *y*+1/2, −*z*+1/2.
